# Vehicle Position Estimation Based on Magnetic Markers: Enhanced Accuracy by Compensation of Time Delays

**DOI:** 10.3390/s151128807

**Published:** 2015-11-13

**Authors:** Yeun-Sub Byun, Rag-Gyo Jeong, Seok-Won Kang

**Affiliations:** Korea Railroad Research Institute, 176 Cheoldo bangmulgwan-ro, Uiwang, Gyeonggi-do 16105, Korea; E-Mails: ysbyun@krri.re.kr (Y.-S.B.); rgjeong@krri.re.kr (R.-G.J.)

**Keywords:** localization, magnetic marker, magnetic sensing system (MSS), peak detection, vertical magnetic field (VMF)

## Abstract

The real-time recognition of absolute (or relative) position and orientation on a network of roads is a core technology for fully automated or driving-assisted vehicles. This paper presents an empirical investigation of the design, implementation, and evaluation of a self-positioning system based on a magnetic marker reference sensing method for an autonomous vehicle. Specifically, the estimation accuracy of the magnetic sensing ruler (MSR) in the up-to-date estimation of the actual position was successfully enhanced by compensating for time delays in signal processing when detecting the vertical magnetic field (VMF) in an array of signals. In this study, the signal processing scheme was developed to minimize the effects of the distortion of measured signals when estimating the relative positional information based on magnetic signals obtained using the MSR. In other words, the center point in a 2D magnetic field contour plot corresponding to the actual position of magnetic markers was estimated by tracking the errors between pre-defined reference models and measured magnetic signals. The algorithm proposed in this study was validated by experimental measurements using a test vehicle on a pilot network of roads. From the results, the positioning error was found to be less than 0.04 m on average in an operational test.

## 1. Introduction

Precise estimation techniques for the position and heading angle have been widely utilized for the automation of factories and port logistics systems. In particular, large commercial vehicle manufacturers (e.g., Mercedes Benz, BMW, Volvo, GM, *etc.*) and information technology (IT) companies (e.g., Apple, Google, *etc.*) are increasing their investment in the competitive information and communications technology (ICT) field, as the need for more reliable and cost-effective autonomous driving technologies increases. The final goal is to develop fully automated vehicles to provide passengers with demand-responsive, door to door (D2D) transportation services without time delays, even in harsh environments. To achieve this goal, the vehicle position must be exactly monitored in real-time during its autonomous operation.

To date, numerous attempts have been made to develop reliable and robust tracking of vehicle position, which has been realized with the advancement in sensing techniques. Thus far, various sensor-fusion systems based on global positioning system (GPS) [1,2,3,4], optical devices (e.g., cameras, laser scanners) [4,5,6], and Magnetic Sensing System (MSS) [7,8,9] have been used as integrated modules both to increase reliability and robustness, and to exploit the advantages of each system. GPS is the most commonly used technique for identifying a vehicle’s absolute position in real-time; however, GPS is not usable in indoor environments (or its use is restricted because of interference caused by large concrete structures). Additionally, the visualization technique has recently attracted attention because of its ability to provide important information (including terrain and planimetric features) regarding the area around a vehicle. However, current visualization devices remain expensive, and the development of mapping algorithms adaptive to changes in operating conditions (e.g., obstacles, weather conditions, *etc.*) is a major impediment in most research.

The absolute position is determined by directly identifying the vehicle’s absolute location (e.g., using GPS) or transforming the relative positional information obtained by a sensing device (e.g., using an MSS). In particular, magnetic-marker-based self-positioning systems have several advantages, as follows: independence of weather conditions, low maintenance requirements, and simple construction on existing road infrastructure [9]. Most importantly, these systems are most efficient when used on a planned path, and unlike other sensing technologies, their application is not limited by harsh environments including the inside the buildings or near-surface tunnels. The California Partners for Advanced Transit and Highways (PATH) program successfully demonstrated automatic guidance of a heavy-duty vehicle (e.g., a bus). [10] Additionally, the system’s technical and economic feasibility was proven by a successful trial of a personal rapid transit (PRT) system prototype (*i.e.*, 2getthere), conducted in Masdar city in the UAE [11].

The absolute position of an autonomously operating vehicle is estimated from the relative position information of magnetic markers from a sensor module installed on the vehicle. The estimation accuracy in this method depends on the methodological robustness of the algorithm for minimization of the errors between the predicted values and the real positions. In general, the intensity distribution of the magnetic signals is characterized by a Gaussian function at the location corresponding to the center of the magnetic marker [9,12]; the local positions of magnetic markers can be estimated after passing the magnetic markers, using Hall-effect sensors inside the module that are distributed as a 1D discrete array. Therefore, estimation errors—resulting from the discrepancy in time and space between the detection time of the peak and the actual peak time—must arise. Therefore, signal processing to transform to the 2D data format should be used to minimize the estimation errors.

In previous studies [7,8,9], automatic driving control systems were implemented using only 1D magnetic signals in the lateral direction collected from magnetic markers. This approach is applicable for low-velocity vehicles at very high sampling frequencies that can overcome errors due to time delays. However, the errors increase—becoming significant—as the vehicle speed increases. Additionally, it is impossible to reduce the sampling time to obtain more data during high-speed driving. Therefore, to address this issue, the development of high efficiency algorithms with low calculation loads and hardware (H/W) configuration is required. In general, the measured signals include noise, which leads to difficulties in peak detection. However, the elimination of noise using filters results in distortion (e.g., time shifts) of the signal. Therefore, the 2D positional information based on magnetic signals measured over time is required, both to compensate for the time delays and to overcome the limitations of the raw data—given in 1D for a specific time.

In this study, the empirical investigation of the design, implementation of H/W and software (S/W), and assessment of the magnetic-marker-based positioning system was carried out for a Korean mini-tram PRT vehicle. In short, the magnetic sensing ruler (MSR) was developed to determine the absolute location of the vehicle by detecting the peak signal corresponding to the actual position of a magnetic marker from the array of signals. In this process, an additional algorithm to compensate for time delays in the signal processing to enhance accuracy despite noise was developed. The signal processing scheme proposed in this study was validated by experimental assessments using a test vehicle (*i.e.*, a modified golf cart) on a pilot network of roads implemented on the grounds of the Korea Railroad Research Institute (KRRI).

## 2. Magnetic Sensing Ruler

### 2.1. Magnetic-Marker-Based Positioning System

**Figure 1:**
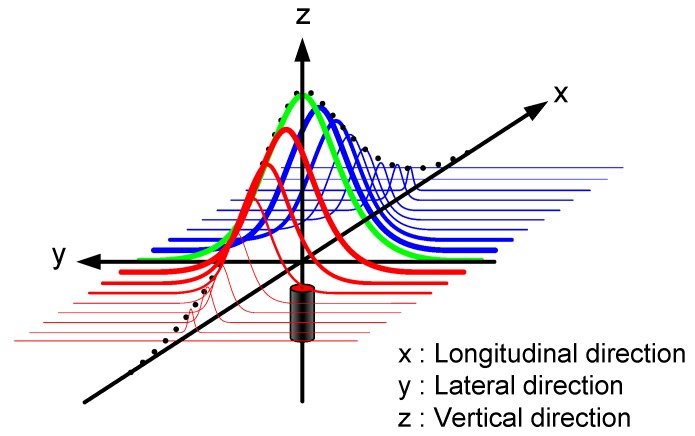
The array of magnetic signals as a series of Gaussian distribution functions in 3D space.

Cylindrical magnetic markers (15 mm in diameter and 30 mm in length) were buried underneath the ground along the test track. For the detection of magnetic fields emanating from the markers, the MSR is mounted on the rear bumper (e.g., approximately 0.08 m above the ground), parallel to the axle axis. As depicted in Figure 1, it is expected that the strengths of the magnetic signals detected using each sensor in the MSR have a Gaussian distribution around the center of the magnetic marker. The height of the peak is most prominent at the moment at which the MSR is passing directly above the magnetic marker. Thereafter, the strength of the signal gradually becomes weaker.

**Figure 2:**
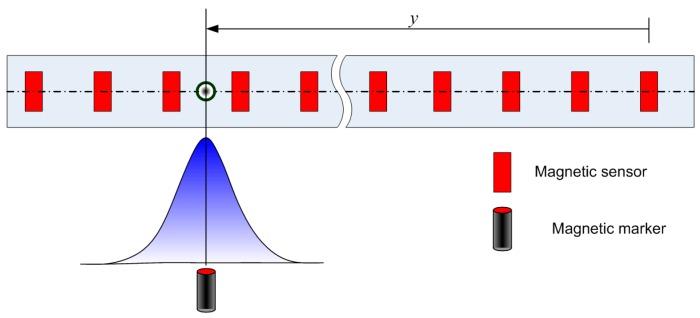
Simple schematic of multi-sensor array in the MSR.

The MSR periodically (e.g., approximately every 30 ms) measures the magnetic signals while the vehicle is autonomously driving on the road. During vehicle operation, the MSR has the role of determining the absolute location of the vehicle by detecting the peak signal corresponding to the actual position of a magnetic marker from the array of signals. The multi-sensor array is arranged with a constant gap (*i.e.*, 48 mm) as shown in Figure 2. The strength of the magnetic signal measured by each sensor is collected and sent to the signal processor, which performs peak detection.

**Figure 3:**
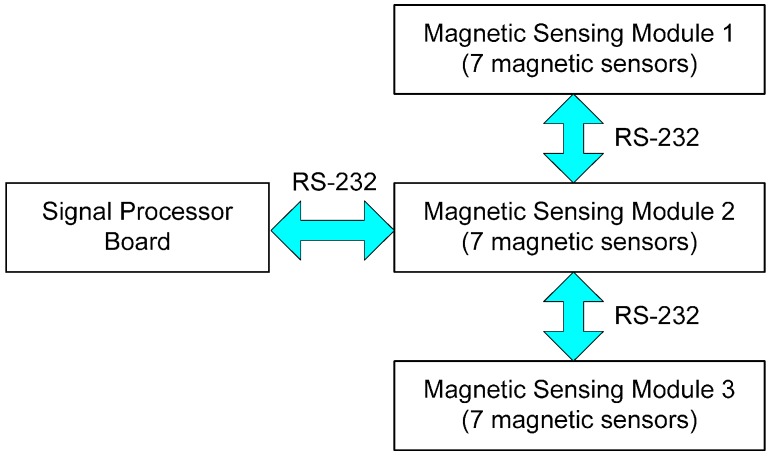
Design of MSR and data flow of magnetic signals.

The completely assembled MSR controller board was integrated with three modular boards—each of which has an array of seven Hall-effect sensors for ease of maintenance and design, and based on the width of the vehicle. The data collected by each modular board is sent to the signal processor (*i.e.*, an MCU) via RS-232 communication links at the time of request by a vehicle host computer, as shown in Figure 3. The host computer performs both calculation of the absolute position and orientation of the vehicle, and actuation for autonomous driving. The position and orientation are identified by referring to real-time information regarding the vehicle status collected by the MSR and from other sensors such as gyroscopes, steering angle sensors, and wheel encoders. The signals measured by the gyroscope and the wheel encoders are used for estimating the posture variation of the vehicle by calculating the moving distance and the angular velocity based on the kinematic model during the sampling time. In addition, the steering angle sensor is dedicated for steering control in vehicle guidance.

### 2.2. Design and Fabrication of MSR

**Figure 4:**
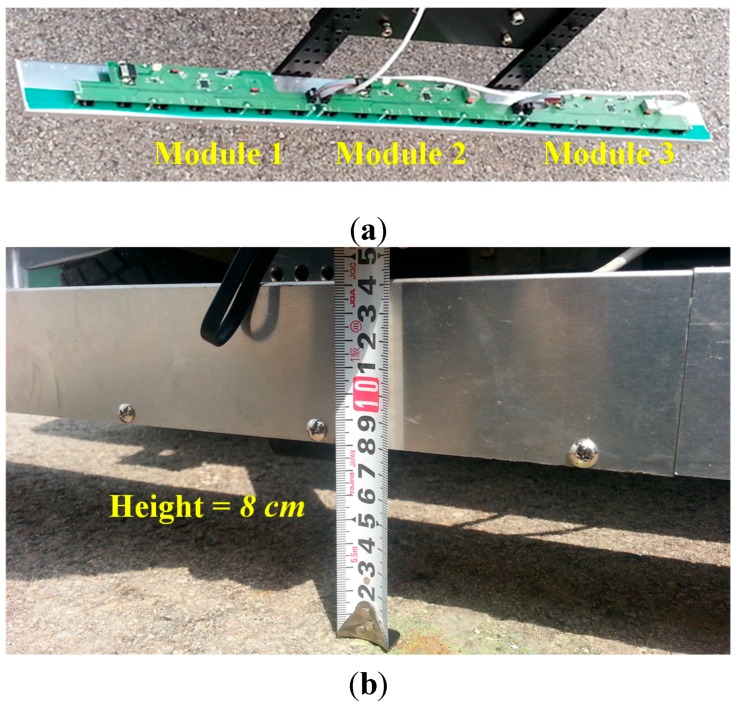
Fabrication and installation of magnetic sensing array: (**a**) Assembly of three modular boards; (**b**) MSR mounted on the vehicle.

AMI302 devices (procured from Aichi Steel, Tokai-shi, Aichi, Japan) were used for measurement of the magnetic field strength (MFS). These sensors provide 3D positional information by integrating the data from the orthogonal magneto-impedance (MI) sensing elements. The magnetic sensing modules—each with a seven-sensor array—were connected next to each other. The data collected by the 21 sensors in the MSR are sent to the main controller (*i.e.*, the vehicle host computer) via the signal processing board (*i.e.*, the MCU), as shown in Figure 3. The assembled MSR shown in Figure 4 was mounted on the rear bumper at a height of approximately 8 cm above the ground, parallel to the rear axle of the test vehicle (*i.e.*, a modified golf cart).

## 3. Peak Detection Algorithm

**Figure 5:**
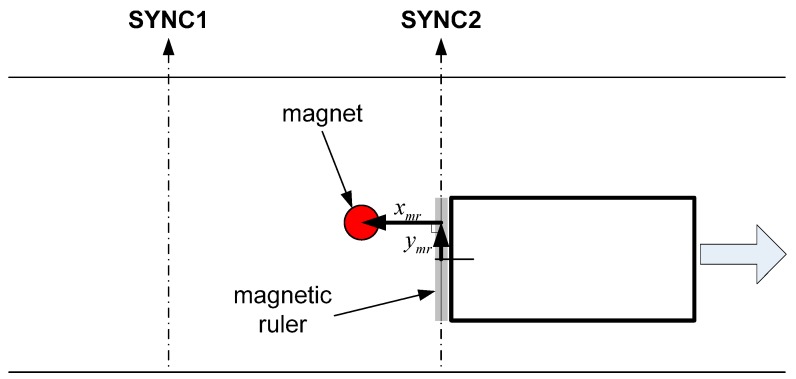
Vehicle local Cartesian coordinate system to determine the relative position of the magnetic markers based on peak detection by the MFS.

**Figure 6:**
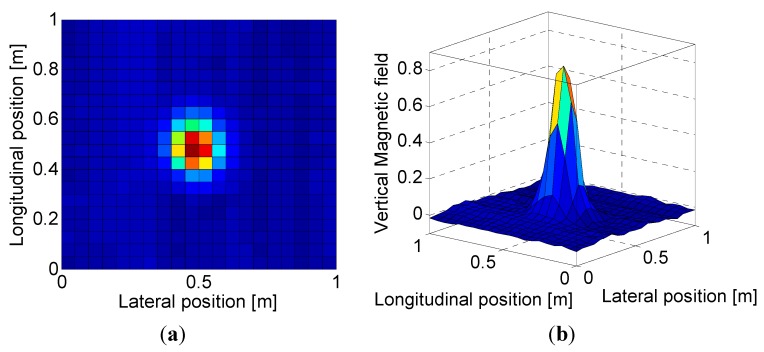
Distribution plot from MFS: (**a**) In 2D; (**b**) In 3D.

In the magnetic-markers-based absolute position estimation method, it is very important to detect the center position of the magnetic markers on the road while driving. In general, the MFS measured by the MSR has the form of a Gaussian function at the location corresponding to the center of the magnetic marker. Because the sensors in the MSR are installed perpendicular to the driving direction of the vehicle—as shown in Figure 4a—the MFS obtained by the MSR has the form of 1D spatial data at a given time. Therefore, the center position of the magnetic marker can be determined by evaluating the variations in slope due to the attenuation of MFS immediately after passing a peak in the Gaussian distribution of the MFS. Therefore, a time discrepancy between peak detection (*i.e.*, SYNC2 in Figure 5) and the actual peak occurrence (*i.e.*, the red circle in Figure 5) arises, which necessitates compensation of time delays resulting from the signal processing time. For this purpose, the 1D spatial data series monitored over time is converted into 2D positional data—as shown in Figure 6a—via the time-to-space transformation algorithm proposed in this study.

The strengths of the magnetic field in 3D [Figure 6b] have a symmetric “bell curve” shape; the height of the curve’s peak depends on the distance between the sensors and the magnetic markers. The center point in the 2D positional information—depicted as a contour plot in Figure 6a—indicates the actual position of the magnetic marker on the planned path. Additionally, the 2D contour plot is obtained by analyzing the changes in the strength of the magnetic signals measured over time during operation of the vehicle. In this process, the time-domain 1D spatial data collected during travel between two magnetic markers are transformed into 2D spatial-domain data.

### 3.1. Modeling of Vertical Magnetic Field

**Figure 7:**
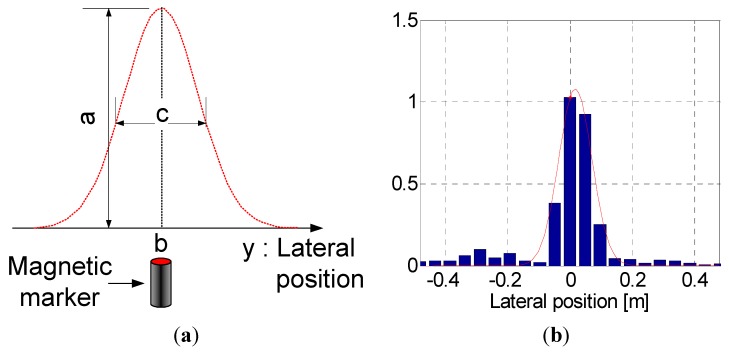
Gaussian function to characterize the MFS at the center of the magnetic marker: (**a**) Mathematical definition of a Gaussian function; (**b**) 1D discrete data measured by the sensor array.

Assuming that the sensor is directly above the magnet and that the distance to the magnet has a known value (e.g., 0.08 m), the reference Gaussian function (given in Figure 7a) for modeling of the vertical magnetic field (VMF) is mathematically expressed as follows:

(1) $$f(y)=a\;\text{exp}\left(-\frac{{\left(y-b\right)}^{2}}{2c^{2}}\right)$$

where *y* is the lateral position in Figure 5, a is the maximum amplitude of the magnetic signal, *b* is the position of the center of the magnet, and *c* is the Gaussian RMS (root mean square) width. In a sample test, the values of *a* and *c* were found to be approximately 0.84 and 0.12, respectively, at a distance of 0.08 m between the sensor and the magnetic marker.

The point at which the errors between the reference model in Equation (1) and the measured values are minimized is defined as the center of the magnet. Figure 7b presents a comparison of the measured values using the MSR with the reference model; this comparison confirms that the measured data closely matches the Gaussian profile. In addition, we found that only sensors within a distance of 0.15 m (in radius) from the center of the magnet could detect the magnetic fields. The intervals between magnetic markers buried along the path are approximately 3 m for straight and 2 m for curved roads, respectively. Thus, the interference between magnetic fields from adjacent markers can be excluded in this study.

### 3.2. Lateral Position (y_mr_) Estimation of Magnetic Markers

**Figure 8:**
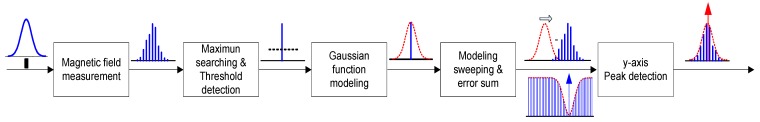
Signal processing flow chart for peak detection in the *y* axis (lateral direction of MSR).

The strength of the magnetic signal is measured by each sensor at every sampling time. The collected data sequence indicates the MFS at each sensor in the lateral direction (the *y* axis in Figure 5). The center of the VMF is determined by comparing the 1D discrete profile of the MFS around each sensor to the reference Gaussian model. That is, the location at which the difference is minimal is regarded as the position of the magnetic marker. Figure 8 presents the schematic procedure for signal processing to identify the position of the peak intensity (*i.e.*, the location of a magnetic marker) in the lateral direction. The validity of the measured MFS is confirmed if the peak intensity is higher than the threshold level, which is determined by investigating the minimum level of MFS required for detection of the magnetic markers:

(2) $$f_{\text{max}}=\text{max}\left(\left|F\right|\right)$$

where *F* = [*f_1_ f_2_* ⋯ *f_n_*], *n* is the number of the sensors, and *f_max_* is the maximum value of the MFS.

Once the MFS data is detected by the MSR at a sampling time, the parameters *a* and *c* in Equation (1) can be determined so as to define the reference Gaussian profile based on the point at which the maximum intensity (*f_max_*) is observed. Then, the similarity of the measured data set (*F*) to the reference model at a constant interval (e.g., *dy* = 0.005 mm) is evaluated for all sections of the MSR (*Y_c_*). The examination interval (*dy*) is determined by both calculation loads and system requirements, so as to increase the precision of position estimation:

(3) $$Y_{c}=\left[\begin{array}{cccc} y_{c0} & y_{c1} & \cdots & y_{ck} \end{array}\right]=\left[\begin{array}{cccc} 0 & dy & \cdots & kdy \end{array}\right]$$

where *k* = (*L*/*dy*) − 1, where *L* denotes the total length of the MSR.

To find the exact *y* position of the peak intensity, the center point of the reference Gaussian function is placed at each examination point while sweeping through the entire range of the MSR. Then, the discrete intensity data set ($\bar{F_{j}}$) is obtained as a function of the sensor position (*Y*):

(4) $${\bar{F}}_{j}=f_{\text{max}}\;\text{exp}\left(-\frac{{\left(Y-y_{cj}\right)}^{2}}{2\sigma^{2}}\right),j=0,1,\cdots ,k$$

where *Y* = [*p_0_ p_1_* ⋯ *p_n − 1_*] = [0 *dp 2dp* ⋯ (*n* − 1)*dp*] and *dp* denotes the distance (i.e., 0.048 mm) between sensors in the MSR. Finally, the relative position (*y_mr_*) of the peak from the center of the MSR is defined as the location (*i.e.*, Equation (5)) at which the summation of errors (*e_yj_*) between $\bar{F_{j}}$ at each examination point and *F* are at a minimum; its mathematical expression is given by Equation (6):

(5) $$\left(i_{y\;\text{min}},\varepsilon_{y\;\text{min}}\right)=\text{min}\left(\left|E_{y}\right|\right)$$

(6) $$\begin{array}{c} with\;E_{y}=\left[\begin{array}{cccc} e_{y0} & e_{y1} & \cdots & e_{yk} \end{array}\right]\\ e_{yj}=\sum \left|{\bar{F}}_{j}-F\right|,j=0,1,\cdots ,k\\ y_{mr}=L/2-i_{y\;\text{min}}\times dy \end{array}$$

where *i_ymin_* is the index of the examination point with minimum error (*ε_ymin_*).

### 3.3. Longitudinal Position (x_mr_) Estimation of Magnetic Markers

*x_mr_* represents the relative position of the magnetic marker from the MSR at a given sampling time (*i.e.*, SYNC2 in Figure 5). The peak detection in the driving direction (*x* axis in Figure 5) is achieved by observing the attenuation of the MFS immediately after passing the peak point. The MFS profile (e.g., the Gaussian RMS width) in the *x* axis as a function of time varies with the speed of the vehicle. Therefore, to define the peak position in the driving direction, the 1D discrete data of the MFS collected over time should be transformed into 2D spatial data. To achieve the time-to-space transformation, the maximum peak intensity over time is monitored whenever the driving distance satisfies a specified value (*s*). The value of *s* is calculated by integrating the moving distance with a speed *v_t_* during a sampling time (*dt*); it is pre-determined to obtain an adequate reference Gaussian profile based on both the vehicle speed and the detection range of the sensors, so as to obtain an effective MFS (e.g., the value of *s* was approximately 0.02 m in this study.).

To define the reference Gaussian profile, the maximum intensity values (*F_max_*) at each distance interval (*Dis*) and the slope variations (*Slop*) between them are collected until the intensity is completely attenuated after the vertex of the MFS in the driving direction. *F_max_* is the set of maximum intensities at each VMF over time:

(7) $$f{\left(k\right)}_{\text{max}}=\text{max}\left(\left|F\left(k\right)\right|\right)$$

(8) $$F_{\text{max}}=\left[\begin{array}{cccc} f{\left(k\right)}_{\text{max}} & f{\left(k-1\right)}_{\text{max}} & \cdots & f{\left(k-n\right)}_{\text{max}} \end{array}\right]$$

(9) $$Dis=\left[\begin{array}{cccc} s\left(k\right) & s\left(k-1\right) & \cdots & s\left(k-n\right) \end{array}\right]$$

(10) $$Slop=\left[\begin{array}{cccc} slop\left(k\right) & slop\left(k-1\right) & \cdots & slop\left(k-n\right) \end{array}\right]$$

where *slop*(*k*) = *f*(*k*)_*max*_ − *f*(*k* − *1*)_*max*_, *n* is the total number of data acquired to define the reference Gaussian profile in the driving direction, and *k* is the discrete time index at each distance interval *s*(*k*). Peak detection is confirmed if the measured signal indicates success (VMF exceeds the threshold level) and the discrete derivative between VMF is changed to a negative slope. 

**Figure 9:**
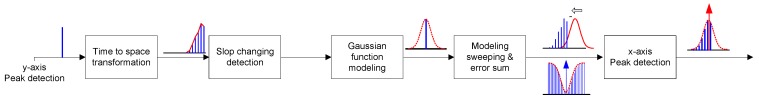
Signal processing flow chart for peak detection using compensation for time delays in the *x* axis (driving direction of the vehicle).

The parameters *a* and *c* in Equation (1) of the reference Gaussian profile in the driving direction (*x* axis in Figure 5) are determined based on the time at which VMF [*f*(*k*)_*max*_] is detected. Then, in a similar manner as for the lateral direction, the differences between the measured data and the reference Gaussian profile are evaluated to determine the exact location of the magnetic marker. To achieve this goal, *F_max_* is converted from a time domain representation to a space (*i.e.*, sampling location) domain representation. Additionally, the maximum intensity value set (*F_max_*) is compared to the reference Gaussian profile [$\bar{F}\left(x_{ci}\right)$] whenever passing each an examination interval (*X_c_*) over the cumulative moving distance (*X*) until the next sampling cycle. Finally, the exact position of the magnetic marker in the driving direction is found, because the location at which the summation of errors between $\bar{F}\left(x_{ci}\right)$ at each examination point and *F_max_* are at the minimum. Figure 9 represents the schematic procedure for signal processing to identify the position of peak intensity in the driving direction:

(11) $$\bar{F}\left(x_{ci}\right)=f{\left(k\right)}_{\text{max}}\;\text{exp}\left(-\frac{{\left(X-x_{ci}\right)}^{2}}{2\sigma^{2}}\right),i=0,1,\cdots ,k$$

(12) $$\begin{array}{ll} X & =\left[\begin{array}{ccccc} 0 & x_{1} & x_{2} & \cdots & x_{n} \end{array}\right]\\ & =\left[\begin{array}{ccccc} 0 & s\left(k\right) & \sum_{i=0}^{1}s\left(k-i\right) & \cdots & \sum_{i=0}^{n}s\left(k-i\right) \end{array}\right] \end{array}$$

where *dx* denotes the examination interval. In Figure 5, the relative position (*x_mr_*) of the peak in the driving direction from the MSR is determined using Equation (15):

(13) $$\left(i_{x\;\text{min}},\varepsilon_{x\;\text{min}}\right)=\text{min}\left(\left|E_{x}\right|\right)$$

(14) $$\begin{array}{lll} with & & E_{x}=\left[\begin{array}{cccc} e_{x0} & e_{x1} & \cdots & e_{xn} \end{array}\right]\\ & & e_{xi}=\sum \left|\bar{F}\left(x_{ci}\right)-F_{\text{max}}\right|,i=0,1,\cdots ,n \end{array}$$

(15) $$x_{mr}=i_{x\;\text{min}}\times dx$$

where *i_xmin_* is the index number of the examination point with minimum error (*ε_xmin_*).

### 3.4. Peak Detection using 2D Positional Information

**Figure 10:**
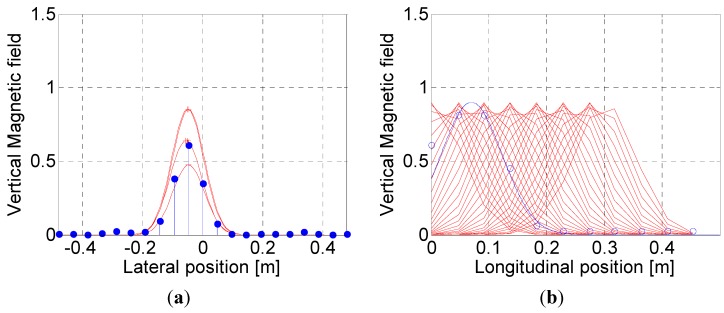

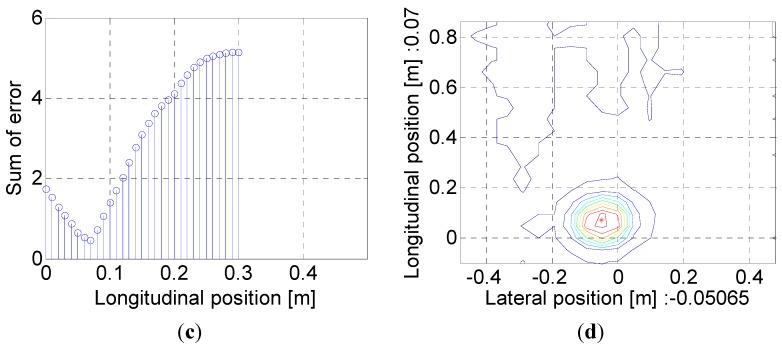
Results of peak detection using the algorithm for compensation of time delays: (**a**) Lateral position detection; (**b**) Longitudinal position detection; (**c**) Error calculations in longitudinal position detection; (**d**) Real-time detection of magnetic markers using 2D positional information.

As mentioned previously, the 2-D positional information of the magnetic markers is given as the relative location (*i.e.*, *x* and *y* values in Figure 5) of the VMF in both the driving and lateral directions of the vehicle’s local Cartesian coordinate system. Figure 10 represents the position estimation results for the magnetic markers, which is carried out using the approach proposed in this study. Figure 10a shows the comparison between the reference Gaussian profile and the measured MFS in the process of finding the lateral position (*y*) of the VMF. Time-to-space transformed signals and their comparison to the reference Gaussian profile in the driving direction are plotted in Figure 10b in which “*0*” in the *x* axis indicates the position of the MSR. We observed that the position of the magnetic marker was located behind the vehicle position, which could be correctly predicted by developing an algorithm to consider the time delays in measurements. Figure 10c is the summation of errors between the reference Gaussian profile and the measured signals at each examination point. The vertex in the 2D contour plot shown in Figure 10d indicates the detected location (*) of the magnetic marker in the vehicle’s local Cartesian coordinate system.

## 4. Localization Based on Magnetic Markers

MFS data measured by the MSR is converted into relative positional information (*x_mr_*, *y_mr_*) with respect to the vehicle local coordinate frame using the VMF detection algorithm. Eventually, the absolute position of a vehicle in the world-fixed coordinate system is obtained through coordinate transformation to a global coordinate system based on relative positional data in the vehicle local coordinate system combined with an odometer reading via an extended Kalman filter (EKF).

### 4.1. Odometry Model

**Figure 11:**
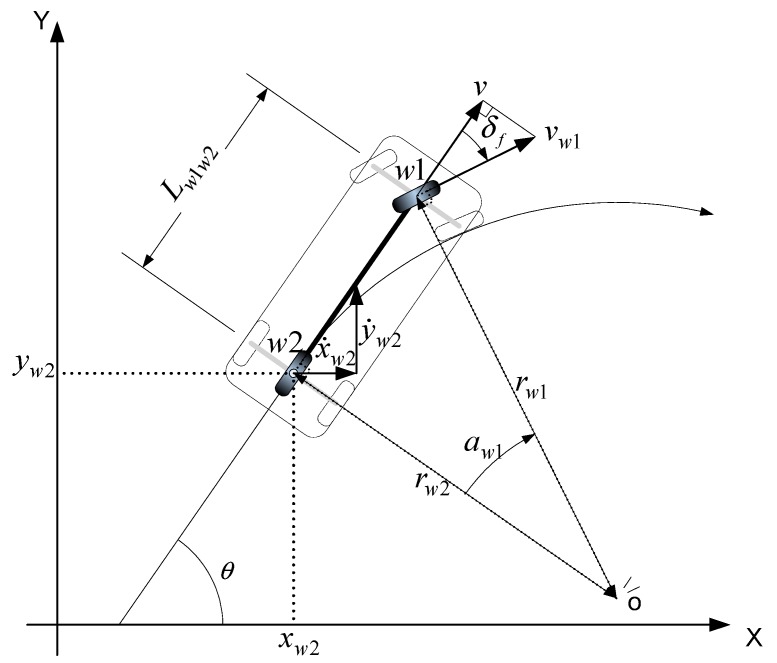
Schematic of vehicle kinematic model.

The modified golf cart was used as the test vehicle for experimental validation of the localization algorithm. The vehicle is equipped with four wheels, but a bicycle model (*i.e.*, using two wheels) was applied to simplify the kinematic model of the test vehicle. Figure 11 shows the movement of a vehicle according to a kinematic model in the global coordinate system. We assume that there is no slippage between the wheel and the road surface, and that the vehicle position is represented by the middle point of the rear axle *w_2_* with Cartesian coordinates (*x*_*w2*(*k*)_, *y*_*w2*(*k*)_) at time *t_k_*. *θ_k_* is the vehicle heading angle at time *t_k_*. Assuming that the vehicle moves along a circular trajectory around a virtual center *O* (*i.e.*, the dotted line in Figure 11) from *t_k_* to *t_k + 1_*, its position and orientation at time *t_k + 1_* are given by:

(16) $$\begin{array}{l} x_{w2\left(k+1\right)}=x_{w2\left(k\right)}+\Delta d_{k}\;\text{cos}\left(\theta_{k}+\Delta \theta_{k}/2\right)\\ y_{w2\left(k+1\right)}=y_{w2\left(k\right)}+\Delta d_{k}\;\text{sin}\left(\theta_{k}+\Delta \theta_{k}/2\right)\\ \theta_{k+1}=\theta_{k}+\Delta \theta_{k} \end{array}$$

where *Δd* is the distance travelled during a sampling interval.

#### 4.1.1. System Model

The system model is mathematically defined by a state vector *X_k_* = [*x_w2(k)_ y_w2(k)_ θ_k_*]^*T*^ and an input vector *u_k_* = [*Δd_k_ Δθ_k_*]^*T*^ as follows:

(17) $$X_{k}=f\left(X_{k-1},u_{k},\gamma_{k},\sigma_{k}\right)$$

where *γ_k_* and *σ_k_* represent the system and input noise with covariance matrices *Q* and *T*, respectively.

#### 4.1.2. Measurement Model

**Figure 12:**
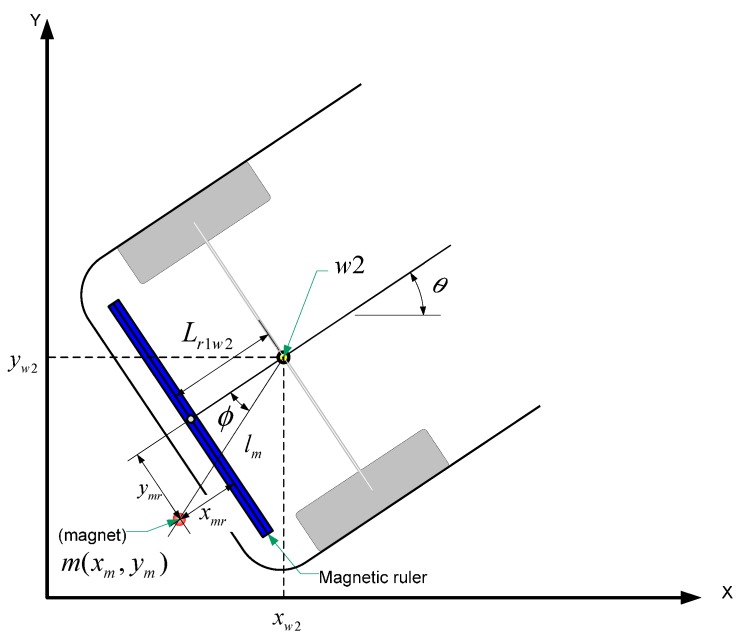
Schematic of measurement model with respect to the central positions of the rear axle and MSR.

The measurement model, which is based on the detection of magnetic markers using an MSR mounted at the rear bumper, in parallel with the rear axle, as shown in Figure 12, is represented by Equation (18). Whenever the magnetic markers are detected by the MSR, the measurements of the distance *l_m_* and the angle *φ* to the detected position of a magnetic marker (*x_m_*, *y_m_*) from the central point (*x_w2_*, *y_w2_*, *θ*) of the rear-wheel axis (*w_2_*) are given by the following nonlinear vector function:

(18) $$h\left(X_{k},k\right)=\left[\begin{array}{c} l_{m}\\ \phi \end{array}\right]=\left[\begin{array}{c} \sqrt{{\left(x_{w2(k)}-x_{m}\right)}^{2}+{\left(y_{w2(k)}-y_{m}\right)}^{2}}\\ {\text{tan}}^{-1}\left(\frac{y_{w2(k)}-y_{m}}{x_{w2(k)}-x_{m}}\right)-\theta_{k} \end{array}\right]$$

Similarly, the nonlinear measurement model can be defined as follows:

(19) $$Z_{k}=h\left(X_{k},k\right)+v_{k}=\left[\begin{array}{c} l_{m}\\ \phi \end{array}\right]=\left[\begin{array}{c} \sqrt{dy^{2}+dx^{2}}\\ {\text{tan}}^{-1}\left(\frac{dy}{dx}\right) \end{array}\right]$$

where *dx* = *L_r1w2_* + *x_mr_*, *dy* = *y_mr_*, *L_r1w2_* is the distance from the central point of the MSR to the central point of the rear axle, the position (*x_mr_*, *y_mr_*) denotes the location of the magnetic marker with respect to the central point of the MSR, and *v_k_* is a zero-mean white noise vector with covariance matrix *R_k_*.

### 4.2. Extended Kalman Filter (EKF) Algorithm

The EKF is used for real-time estimation of the vehicle’s pose (*i.e.*, position and orientation) based on convergence with the odometer data (e.g., gyroscope, steering angle, and wheel encoder) based on the measurement results using the MSR. The EKF predicts the future state of the system using two components: the time update equation and the measurement update equation. The time update and the measurement update are regarded as the prediction stage and the correction stage, respectively.

#### 4.2.1. Prediction Stage

The future state of the system model *f*(·) and the state error covariance matrix *P_k_* are predicted using the time update equations:

(20) $$X_{k}^{-}=f\left(X_{k-1}^{-},u_{k},0,0\right)$$

(21) $$P_{k}^{-}=A_{k}P_{k-1}A_{k}^{T}+B_{k}T_{k}B_{k}^{T}+Q_{k-1}$$

where the system *A_k_* and input *B_k_* are the following Jacobian matrices of the partial differential of the system *f*(·) to *X_k_* and *u_k_*:

(22) $$A_{k}=\frac{\partial f}{\partial x}=\left[\begin{array}{ccc} 1 & 0 & -\Delta d_{k}\;\text{sin}\left(\theta_{k}+\Delta \theta_{k}/2\right)\\ 0 & 1 & \Delta d_{k}\;\text{cos}\left(\theta_{k}+\Delta \theta_{k}/2\right)\\ 0 & 0 & 1 \end{array}\right]$$

(23) $$B_{k}=\frac{\partial f}{\partial u}=\left[\begin{array}{cc} \text{cos}\left(\theta_{k}+\Delta \theta_{k}/2\right) & -\Delta d_{k}\;\text{sin}\left(\theta_{k}+\Delta \theta_{k}/2\right)\\ \text{sin}\left(\theta_{k}+\Delta \theta_{k}/2\right) & \Delta d_{k}\;\text{cos}\left(\theta_{k}+\Delta \theta_{k}/2\right)\\ 0 & 1 \end{array}\right]$$

#### 4.2.2. Measurement Model

The measurement update is computed only when the magnetic markers are successfully detected. The Kalman gain matrix *K_k_*, state estimate *X_k_*, and state error covariance *P_k_* are calculated using the following measurement update equations:

(24) $$K_{k}=P_{k}^{-}H_{k}^{T}{\left[H_{k}P_{k}^{-}H_{k}^{T}+R\right]}^{-1}$$

(25) $$X_{k}=X_{k}^{-}+K_{k}\left(Z_{k}-h\left(X_{k}^{-}\right)\right)$$

(26) $$P_{k}=\left(I-K_{k}H_{k}\right)P_{k}^{-}$$

where *I* is the identity matrix and *H_k_* denotes the Jacobian matrix of the partial differential of *h*(·) for *X_k_*:

(27) $$H_{k}=\frac{\partial h}{\partial x}=\left[\begin{array}{ccc} \frac{x_{\omega 2}-x_{m}}{l_{m}} & \frac{y_{\omega 2}-y_{m}}{l_{m}} & 0\\ \frac{-y_{\omega 2}+y_{m}}{l_{m}^{2}} & \frac{x_{\omega 2}-x_{m}}{l_{m}^{2}} & -1 \end{array}\right]$$

## 5. Results and Discussion

**Figure 13:**
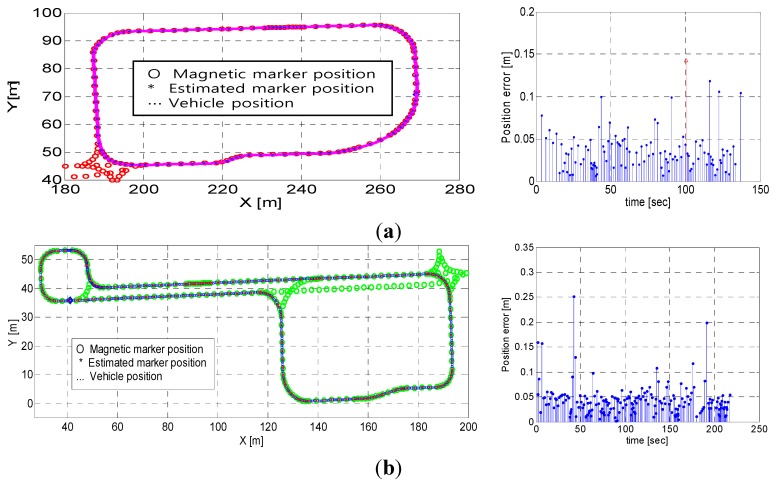
Estimation results (*i.e.*, comparison of estimated positions with absolute positions of magnetic markers and their estimation errors) in practical operation of 2 m/s on average for validation of the algorithm at different loops of the test route with a length of (**a**) 237.96 m and (**b**) 405 m, respectively.

The operational experiments were conducted to verify the performance of the position estimation algorithm proposed in this study. The absolute positions of the test vehicle in autonomous operation were calculated based on the real-time measurements of the MFS using the MSR. From the results shown in Figure 13a, we found that 111 markers were successfully detected, and that only one marker was missed along the test route of approximately 237.96 m in length. The estimated positions of the detected magnetic markers on the planned path are shown in Figure 13a. The symbols “*” and “o” in Figure 13 denote the estimated positions and the actual (or absolute) positions (*i.e.*, the measured data) of the magnetic markers, respectively. The estimation errors were calculated based on the Euclidean distance between the two positions. In essence, the position values estimated by the MSR should coincide with the absolute position if the positions and orientations of a vehicle in operation are correctly calculated. However, in reality, errors between them inevitably exist, because of various factors such as measurement errors of the sensors (e.g., moving distance, angular velocity, and steering angle), shortcomings of the kinematic model (e.g., wheel slip), and signal processing errors (e.g., distortion of magnetic signals and delay by signal processing). The errors in this study were found to be approximately 0.035 m, on average, with a maximum error of 0.118 m. These results are comparable to those obtained in real-time kinematics (RTK) mode of GPS, in terms of precision (*i.e.*, the several-centimeters level) [13]. As shown in Figure 13a, one of the magnetic markers resulted in an invalid detection because of its abnormally high error. Furthermore, the experimental operation at a longer test route is shown in Figure 13b, and 185 markers were successfully detected without missed markers in the entire test route of approximately 405 m in length. The maximum estimation error was 25 cm at a specific magnetic marker, but the average value was at a similar level as in the previous case (*i.e.*, 4 cm).

**Figure 14:**
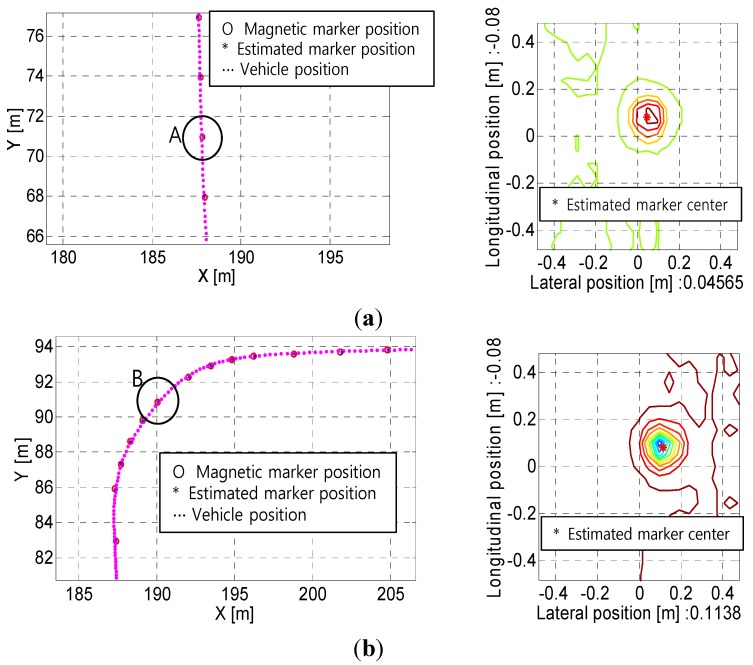
Comparison of estimation results in operation at: (**a**) Straight sections of the route; (**b**) Curved sections of the route.

Figure 14 presents the estimation results for operation on both straight and curved sections of the route. From the results, the MSR system developed in this study exhibited good performance, regardless of the curvature condition of the route. In particular, it was demonstrated that the positions of magnetic markers were successfully estimated, despite the existence of noise and distortion of the magnetic signals.

**Figure 15:**
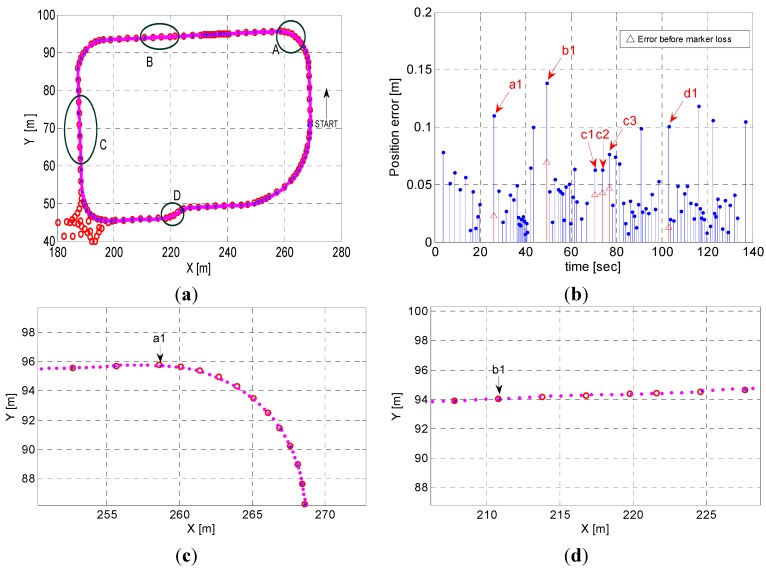

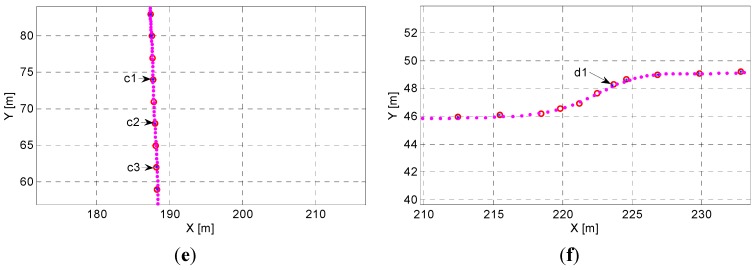
Estimation results with consideration of detection errors at 4 different sections of the test route: (**a**) Comparison of estimated positions with absolute positions of magnetic markers; (**b**) Estimation errors; (**c**) Section A; (**d**) Section B; (**e**) Section C; (**f**) Section D.

Four different sections (*i.e.*, A–D) shown in Figure 15a were defined to consider the effects by the detection errors of the magnetic markers. The magnetic markers were intentionally to be missed in these sections for realization of the detection errors. Figure 15c (*i.e.*, section A) presents the results of the case where it was assumed that the 6 magnetic markers installed on the curved route with 2 m intervals are continuously missed. The real-time position and orientation of the vehicle is estimated by means of the dead-reckoning method based on a kinematic model while the vehicle is not detecting the magnetic markers. Also, in Figure 15d, it was assumed that the four continuous magnetic markers (*i.e.*, section B) on the straight route are missed. In addition, Figure 15e,f present the case missed at every other magnetic marker at the straight route with 6 magnetic markers and at the s-shape curve with four continuous magnetic markers, respectively. From the simulation results, it was observed that the position estimation errors were slightly increased from 6.9 cm to 13.8 cm (maximum errors), as summarized in Table 1. 

**Table 1:** Changes in estimation errors (cm) by considering detection failure of magnetic markers.

Marker Index	Experiment (Figure 13a)	Simulation (with Assumption of Detection Failure)
*a1*	2.2	11
*b1*	6.9	13.8
*c1*	4.1	6.2
*c2*	4.3	6.3
*c3*	4.6	7.6
*d1*	1.2	10

## 6. Conclusions

An empirical investigation of the design, implementation, and evaluation of an MSS for an autonomous vehicle was performed in this study. The MSR exhibited enhanced accuracy in position estimation of magnetic markers by incorporating an additional algorithm (*i.e.*, conversion to 2-D positional information using a time-to-space transformation) for compensation of time delays caused by sampling interval and signal processing. The combination of odometer readings with measurement of magnetic signals using an EKF also proved to be reliable for vehicle self-localization with high precision; accuracies were comparable to those of the conventional differential global positioning system (DGPS). In the operational assessment using the test vehicle on the test track, the maximum estimation error was found to be only several centimeters. This approach will be further developed by repetitive operation at the system level using a prototype vehicle. This study aims at the development of the MSS suitable for urban transit systems such as a PRT vehicle, usually operated at speed of below 30 km/h. Additional investigations for extended application will hence be performed to secure the desired performance in high speed operation of a vehicle in the next step of this study. Besides this, a laser scanner will be combined with the MSS for prevention of collisions with forward obstacles for further advancement.
